# Psychophysiological Effects of Slow-Paced Breathing on Adolescent Swimmers’ Subjective Performance, Recovery States, and Control Perception

**DOI:** 10.3390/jfmk9010023

**Published:** 2024-01-25

**Authors:** Quentin Merlin, Philippe Vacher, Laurent Mourot, Guillaume Levillain, Guillaume Martinent, Michel Nicolas

**Affiliations:** 1 Laboratory Psy-DREPI (EA 7458), University of Bourgogne Franche-Comté, 21000 Dijon, France; michel.nicolas@u-bourgogne.fr; 2 Research Center for Education Learning and Didactics (EA 3875), Faculty of Sports Science, University Brest, 29200 Brest, France; philippe.vacher@univ-brest.fr (P.V.); guillaume.levillain@univ-brest.fr (G.L.); 3 Maison des Sciences de l’Homme de Bretagne, Fe, MSHB, 35043 Rennes, France; 4 SINERGIES, Université de Franche-Comté, 25000 Besançon, France; laurent.mourot@univ-fcomte.fr; 5 Plateforme Exercice Performance Santé Innovation, Université de Franche-Comté, 25000 Besançon, France; 6 Laboratory L-VIS (EA 7428), University of Claude Bernard Lyon 1, 69200 Lyon, France

**Keywords:** SPB-NoHRVB, breathing, adolescent athletes, stress, recovery, subjective training performance

## Abstract

This study examined the effect of a Slow-Paced Breath (i.e., 6 breaths per minute) without Biofeedback (SPB-NoHRVB) protocol on semi-elite adolescent swimmers’ psychological and physiological states during a seven-week ecological training period. A linear mixed-effects multilevel regression analysis approach was used with 13 adolescent national-level swimmers. Athletes were randomly assigned to an intervention group (n = 7) and a control group (n = 6). Seven waves of assessments were completed weekly during a seven-week training preparation in ecological conditions. During the protocol, swimmers completed subjective quantitative measures (RESTQ-36-R-Sport; cognitive perceived stress and control states about the training process, training subjective performance, and subjective internal training load) and physiological heart rate (HR) (HR of exercise, absolute and normalized HR recovery during the first 60 s of recovery; HRR60 and nHRR60) and heart rate variability (HRV) (MeanRR, RMSSD, LFnu and HFnu, LF/HF ration) tests (through a submaximal heart rate (5′-5′ test) once a week. Results revealed that the SPB-NoHRVB protocol significantly predicts biopsychosocial recovery states, cognitive perception of control, and training subjective performance (i.e., a significant effect of the SPB-NoHRVB protocol with the dependent variables simple time trajectories). However, no significant effects were found for biopsychosocial stress scales, cognitively perceived stress, HR, or HRV markers. Our results suggest that SPB-NoHRVB induces simple evolutions over time for crucial variables in athletes’ adaptation to the training process (i.e., cognitive appraisals and biopsychosocial states). In contrast, it highlights that SPB-NoHRVB does not induce better stress states. This specific effect on the resource component is an exciting result that will be discussed in the manuscript.

## 1. Introduction

In 2019, approximately 58 million children and adolescents lived with an anxiety disorder [1]. Indeed, the transition to adolescence is accompanied by rapid, unexpected, and novel experiences in social, cognitive, physical, emotional, and psychological domains and is a critical period in the development of mental health problems [2,3]. In addition, and specifically for young elite athletes, the pursuit of their studies and the demands of their sport add to the challenges of adolescence itself, creating multiple stressors (e.g., sport-specific, academic, and social) [4]. Therefore, stress and recovery management are valuable for preventing nonfunctional overreaching/overtraining and enhancing training benefits.

From a biopsychosocial perspective, stress and recovery are multidimensional and interactive [5,6]. From this perspective, stressors can be sporting and physiological (workload), sporting and psychological (competitions), and academic and psychological (exams), creating an imbalance and cross-dimensional disturbances (e.g., poor performance, restlessness, irritability, disturbed sleep, etc.) [7]. If not correctly counterbalanced by recovery, this imbalanced state can lead to overtraining [8]. Recovery is also seen as a multi-faceted process (e.g., psychological, physiological, social) [9] and as an inter-individual and intra-individual process over time to restore performance capabilities [6]. This means that several strategies and techniques can be implemented since recovery can be physiological (e.g., active recovery, cold water immersion, nutrition, sleep) and/or psychological (e.g., coping strategies, relaxations) [7]. Consequently, the ability to recover is considered an integral part of athletic training and vital for preserving athletes’ resources [10]. Furthermore, monitoring recovery in the context of athletic performance has gained importance in recent years [11]. This is why it is worth offering interventions focusing on how physiological and psychological interactions can improve and monitor stress–recovery states.

From there, the allostatic stress model, and more specifically the concept of allostatic load, explains how, in response to an environmental demand, heart–brain interactions set up physiological, psychological, and emotional adaptation mechanisms leading to the stress–recovery balance [12]. Then, allostasis involves feedback from the whole brain and body through the autonomic nervous system (ANS) and hypothalamic–pituitary–adrenal axis (HPA) to promote adaptation [13].

According to this model, substantial individual differences in how people react to potentially stressful situations are linked to cognitive interpretation [14]. The way individuals interpret situations cognitively aligns closely with the cognitive appraisals described by Lazarus and Folkman [15]. At the heart of individual adaptation lies the psychological process of cognitive appraisal. The initial step, primary appraisal, assesses the personal relevance of a situation, subsequently leading to the sensation of stress. This is swiftly followed by a secondary appraisal, which gauges an individual’s internal and external capabilities to handle the situation. It is this secondary appraisal that culminates in the feeling of control over stressors. This sense of control can be articulated as the extent to which an individual believes they can dictate their actions and internal feelings, shape their surroundings, or achieve desired results [16]. Another vital element that shapes an individual’s reaction to stress is their physiological state.

Brain–heart interactions, governed by the sympathetic (SNS) and parasympathetic (PNS) nervous system branches of the ANS [17], involve a dynamic balance rather than antagonism [18]. In simple terms, the SNS triggers the “fight or flight” response (increasing heart rate), while the PNS restores resources (decreasing heart rate) [17]. This simultaneous functionality is essential for a healthy organism [18]. Heart rate variability (HRV) measurement allows non-invasive tracking of this balance [19]. A low HRV at rest indicates SNS over-activation and inadequate cardiovascular adaptation, suggesting a disturbed ANS, whereas elevated HRV signals an effectively operating ANS, particularly heightened parasympathetic activity [20]. The vagus nerve’s afferent pathways influence psychological factors by sending signals to the brain, impacting frontocortical and motor cortex activity, attention levels, motivation, perceptual sensitivity, and emotional processing [20].

Considering these interactions, McCraty and Childre (2011) introduced the term physiological coherence, denoting order, harmony, and stability in rhythmic activities within living systems [21]. They proposed that heart rhythm coherence is reflected in the HRV power spectrum by an increase in power in the low-frequency (LF) band and a decrease in power in the very-low-frequency (VLF) and high-frequency (HF) bands [21]. The coherence model suggests that HRV, mediated by vagal efferent fibers, reflects autoregulation capacity [18]. In pleasant emotional states, HRV is coherent and wide, while in unpleasant emotions, it becomes chaotic and narrow [21]. HRV coherence aims to shift unhealthy mental, emotional, and behavioral reactions by changing the afferent input pattern (i.e., cardiovascular system) [22,23]. Laborde et al. (2021) distinguished Slow-Paced Breath with HRV Biofeedback (SPB-HRVB) or without HRV Biofeedback (SPB-NoHRVB) when breathing at six breaths per minute, inducing a coherent resonance frequency in various physiological signals [24]. SPB in population samples showed enhanced autonomic, cerebral, and psychological flexibility, increased HRV linked with emotional control and well-being, and decreased emotional arousal [25,26,27]. In athlete samples, voluntary SPB brought positive psychological improvements, enhanced executive functions, improved sports performance, and had preventive and rehabilitative properties, including improved lung capacity, sleep quality, and athlete well-being during COVID-19 [28,29]. Specifically for recovery, SPB may improve cardiac variability during short-term effort recovery and enhance the perception of recovery and physical exertion [30].

Young athletes can be engaged in dual careers, i.e., pursuing school or university studies simultaneously with their athletic training [31]. Studies of athletic training among student–athletes have shown that this population is subject to high demands at all levels of athletic and non-athletic development. Moreover, [32] emphasizes the importance of providing strategies to help young athletes develop psychological strategies to cope with and manage multiple demands. All these factors encourage us to find applied protocols to help athletes face their difficulties, with a schedule that concedes very little room for heavy-handed protocols. Therefore, the primary goal of this six-week protocol is to evaluate the effects of SPB-NoHRVB (i.e., breathing at six breaths per minute) on the biopsychological states of stress recovery, cognitive appraisals, subjective training performance, perceived exertion, heart rate (HR) (HR of exercise, HRex; absolute and normalized HR recovery during the first 60 s of recovery, HRR60 and nHRR60), and HRV markers (RMSSD, LFnu and HFnu, LF/HF ratio, and MeanRR). From a practical standpoint, the aim is to propose a simple method based on a simple and quick-to-implement tool that can provide solutions in supporting athletes’ biopsychosocial states and stimulating the parasympathetic branch of the ANS. We hypothesize that the intervention group will show (1) lower scores of cognitive and biopsychosocial stress, subjective internal training load, HRex, and sympathetic HRV markers, while (2) higher scores of perceived control, biopsychosocial recovery states, subjective training performance, HRR60, nHRR60, and parasympathetic HRV markers than the no-treatment group.

## 2. Materials and Methods

### 2.1. Population

Thirteen French semi-elite adolescent swimmers, classified based on the criteria by Swann et al. (2015) (i.e., with achievements ranging from medalists to participants in the French Championship in their category) [33], were randomly divided into an experimental group (n = 7; females = 4, males = 3, Age = 13.9 ± 0.38, Height = 164.9 ± 3.97, Weight = 51.71 ± 5.67, YearsExperience = 5.7 ± 0.48) and a no-treatment control group (n = 6; females = 4, males = 2, Age = 13.5 ± 0.55, Height = 164.9 ± 3.9, Weight = 51.17 ± 3.76, YearsExperience = 5.5 ± 0.54). All the swimmers were in the same training group. Thus, the same academic and training program applied to all swimmers. They trained for 16 h per week (specific training = 13 h; physical preparation = 3 h). Finally, to obtain an overview of the athletes’ mental health, the researchers asked whether the athletes were seeing or had seen a psychologist. None of the athletes were concerned. These data were collected orally and individually. Given the ecological nature of our sample, a few additional recommendations are necessary (e.g., availability of population, a priori power analysis) [34]. Since the sample encompasses the entirety of available semi-elite athletes for our study, it is inherently justified to utilize all the available data in line with the principle articulated by Lakens [34]. Then, a priori power analysis was performed using Power IN Two-Level Designs software, which is designed to estimate standard errors of regression coefficients in hierarchical linear models for power calculations [35]. If α is chosen at 0.05, a medium effect size of 0.50 is what we expect, and a power of 0.80 is desired [36], then a sample of 13 participants along seven measurement points is required.

This study was part of the ASDP project validated by the ethics committee of the Alliance Universitaire Bretagne under the number 2303077 and was carried out following the Declaration of Helsinki. After verbal and written explanations of the study, all the subjects gave their written informed consent to participate. For minors, parents/guardians gave full written informed consent. At any time, participants were free to withdraw from the study.

### 2.2. Materials

#### 2.2.1. Biopsychosocial Recovery–Stress States

The short French version of the Recovery–Stress Questionnaire for Athletes (RESTQ-36-R-Sport) was used to measure the recovery–stress state of the athletes [37,38]. We used the general, specific, and total scores of stress and recovery in order to adopt a holistic perspective of the athletes’ recovery and stress states. The response scale asked participants to rate the frequency of each item over the preceding three days/nights on a scale of 0 (never) to 6 (always). Cronbach alpha ranged from 0.74–0.89. Please refer to Table 1 for the complete descriptive statistics.

#### 2.2.2. Cognitive Appraisals—Perceived Stress and Control

An adaptation to the sporting context of the Mastery Scale (MS) [39] was used to assess the level of perceived control, as was an adaptation of the Perceived Stress Scale (PSS) [40]. For a short and quick administration, we used the six French items validated by Martinent and Nicolas (2017) and reworded “competition” into “training” to refer to the training context and not to the competition context (e.g., “I feel able to cope with the stress of the training”; “I have the resources to cope with training pressures”; “I feel able to master the challenges that I could meet during training” [41]. The scores indicated the extent to which the athletes agreed with these statements on a six-point Likert scale (1 = strongly disagree to 6 = strongly agree). Cronbach alpha ranged from 0.80 to 0.86. Please refer to Table 1 for the complete descriptive statistics.

#### 2.2.3. Training Load and Performance

Throughout the study, internal subjective training load (sRPE) and external objective training load (kilometers; KM) were computed. sRPE was calculated as a product of session duration (in minutes) and session intensity using the original Borg’s Rate of Perceived Exertion scale (i.e., 6–20) through the method of Foster et al. (2001). Participants were asked to rate their perceived session exertion (RPE) 30 min after each swimming and physical training session. As swimmers regularly complete two or more training sessions per day, the sRPE, expressed in arbitrary units, was summed for each day to create a daily internal training load. Then, the total sRPE was calculated according to Foster et al. [42]. Additionally, we computed the objective training load, which was calculated as a product of the session volume in kilometers (KM) for each week of training performed by the swimmers. Subjective performance represents the overall swimmers’ satisfaction with their training week. To limit the number of scales and quantitative tools for athletes, the subjective training performance was assessed using a modified version of Borg’s Rate of Perceived Exertion scale (i.e., 6–20) used for sRPE monitoring. In this modified version, 6 corresponds to a very, very poor performance, and 20 to a very, very good performance.

#### 2.2.4. Heart Rate Variability and Heart Rate Recovery

Physiological measurements of HRV were recorded with the Suunto t6 Memory Belt (Suunto, Vantaa, Finland). Swimmers performed a standardized 5 min sub-maximal running exercise at 9 km.h^−1^, followed by 5 min passive recovery (5′- 5′) [43]. The 5′- 5′ test is designed to simultaneously measure sub-maximal heart rate exercise, heart rate recovery, and HRV, which are recognized measures of cardiovascular fitness [44] and cardiac autonomic activity [43]. The data were then imported into Kubios Standard software (Kuopio, Finland, Version 3.5.0) to obtain the following physiological markers:

Heart rate markers:

- Sub-maximal heart rate exercise (HRex): mean HR over the last minute of the submaximal running test.
- Heart Rate Recovery: absolute and normalized HR recovery during the first 60 s of recovery (HRR60 and nHRR60) of the cessation of the submaximal running test. nHRR60 was obtained by calculating [(HRex-HR60)/HRex].

Heart rate variability markers (considering the last 2 min of the 5 min of recovery):

- Frequency Domain
  ○ LFnu and HFnu: the power of the low frequency (LF) and high frequency (HF)
  ○ LF/HF ratio: we calculated the low (LF; 0.04–0.15 Hz) to high (HF; 0.15–0.40 Hz) frequency ratio (LF/HF). This ratio is understood within the context of autonomic balance, recognizing that both sympathetic and parasympathetic activities can simultaneously influence LF power. A low LF/HF ratio often suggests greater parasympathetic activity, while a high ratio may indicate sympathetic dominance or parasympathetic withdrawal [18].
- Time Domain
  ○ RMSSD: It is the root mean square of successive RR interval differences. The RMSSD reflects the beat-to-beat variance in HR and is the primary time domain measure used to estimate the vagally mediated changes reflected in HRV. By capturing PNS modulation, RMSSD reflects both training- and non-training-related stress and can be indicative of positive or maladaptive responses to training demand. RMSSD is also a relevant measure because it is poorly affected by respiration at rest [45]. We chose to use RMSSD because it predominantly captures vagal activity and consistently demonstrates as much reliability as other spectral measures [46].
  ○ MeanRR: The meanRR Interval is the average R-R interval duration in a measurement.

Please refer to Table 1 for the complete descriptive statistics.

### 2.3. Experimental Design

**Figure 1:**
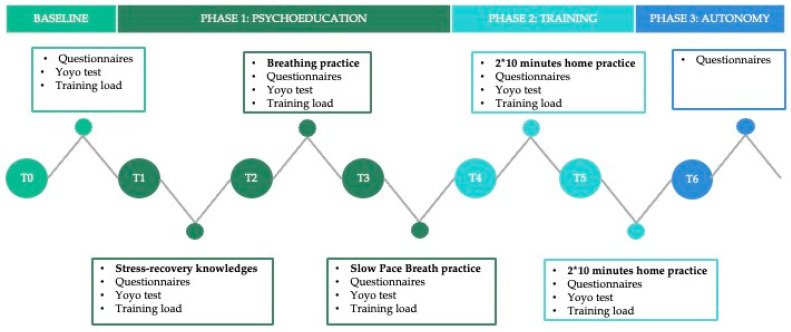
Protocol sequences and summary.

Before the initiation of the SPB-NoHRVB protocol, a clear baseline measurement was established for all participants in both groups (Time 0 of the protocol) one week before the beginning of the protocol. This baseline measurement involved administering the same state questionnaires, such as the REST-Q-36-R-Sport, that were used during the six-week protocol (see Figure 1). The protocol itself lasted six weeks (T1 to T6) and was composed of three phases (i.e., Phase 1—weeks 1 to 3: psychoeducation in a “classroom” condition, SPB; Phase 2—weeks 4 to 5: training twice in environmentally controlled conditions and in daily life; and finally, Phase 3—week 6: learning phase in autonomy). Every Saturday morning, for both the no-treatment group and the SPB-NoHRVB group, physiological measures were taken around one hour before the swimming session, whereas psychological assessments were submitted in a room 30 min after the training session. A T6 measurement for HRV and training load is not included, as it serves to assess subjective variables. All those measures were taken under the supervision of the first author. For the experimental group, the first three weeks (i.e., the psychoeducation phase) consisted of a 30 min psychoeducation and practice session per week. The article’s first author, a mental coach, delivered these sessions. The first session aimed to review the process, the protocol’s key dates, what recovery is, and how it relates to performance. The second session highlighted the links between breathing, the heart, homeostasis, and recovery, followed by breathing practice. The last session consisted of guided breathing practice, downloading and setting up the “breath” app, practicing, and giving advice on breathing at six cycles per minute using the app. Throughout the various supports used during psychoeducation, it was clearly suggested that breathing can promote psycho-physiological recovery and stress states. The emphasis on this point was intended to improve adherence and receptiveness to the program. For the following two weeks (i.e., training phase) and twice ten minutes a day (i.e., once when waking up and once before asleep), the experimental group had to practice breathing at six cycles per minute with the “breath” app. During this phase, the mental coach supervised the exercises, could help the athletes individually if any of them were experiencing difficulties, and ensured that the training was carried out. Finally, during the last week (i.e., the learning phase), the mental coach could no longer interfere, and the athletes were no longer required to perform the breathing exercises. The aim was to see whether the effects of breathing practice were maintained and/or learned from. For details of the protocol’s contents, please refer to Table 2.

### 2.4. Statistical Procedure

Hierarchical Linear Modeling or Multilevel Growth Curve Analysis (MGCA) was utilized to examine the linear trajectories of athletes’ recovery–stress states, cognitive appraisals, and HRV/HR variables [47]. Analyses were carried out with the lme4 package in R [48]. We performed distinct analyses for each psychological metric (i.e., general, specific, and total scores of stress and recovery; perceived control and stress, subjective training performance, and internal training load) and physiological (i.e., HRex, HRR60, nHRR60, LFnu, HFnu, LF/HF, RMSSD, MeanRR) states. Multilevel models extend multiple regressions to nested data (hierarchically structured data). Specifically, repeated measurements (Level 1 units of analysis) were nested within individuals (Level 2 units of analysis). Multilevel models are a flexible approach that can be applied to evaluate inter-individual differences in intra-individual changes over time (i.e., each participant has their own curve). Thus, by taking into account the hierarchical structure of the data, multilevel models provide unbiased estimates of the parameters [47]. Firstly, a series of two-level models estimated the average growth and the individual differences in growth. At Level 1, time (linear trajectory) was entered as a predictor to estimate the average intercept (β0). The intercept reflects the athletes’ initial state. Each model incorporated the random effects for both the intercept and linear slope. Furthermore, our study aimed to investigate the intervention’s impact on the time trajectory for subjective training performance, recovery–stress states, cognitive appraisals, and physiological variables. Consequently, we integrated the interactions of time with the group for the trajectories of the dependent variables. A significant interaction suggests an effect of the intervention (see Table 3).

## 3. Results

### Growth Curve Models Interaction with Group

We performed an in-depth analysis of the time*group interaction (experimental vs. no-treatment control group) across several measures. This aimed to identify if changes during the study differed between groups. A significant interaction suggests varying effects of the protocol over time between groups.

We observed a significant effect for specific recovery (β = 0.19, *p* < 0.10) and a significant one for total recovery (β = 0.16, *p* < 0.01), indicating improved recovery states in the experimental group. In terms of cognitive appraisals, a significant time*group interaction was noted for perceived control (β = 0.12, *p* < 0.01), reflecting enhanced control perception in the experimental group. The subjective training performance model showed a significant time*group interaction (β = 0.64, *p* < 0.01), with the experimental group reporting higher subjective training performance.

However, no significant changes were found in HR and HRV markers. The detailed results are presented in Table 3.

## 4. Discussion

This research aimed to investigate the effects of a 6-week SPB-NoHRVB protocol on the biopsychosocial states of stress–recovery, cognitive appraisals, subjective training performance, and physiological HR and HRV markers in adolescent swimmers. The results indicated that the experimental group, which underwent SPB-NoHRVB, showed higher scores of biopsychosocial recovery, perceived control, and subjective performance than the non-treatment control group.

Perceived control and subjective training performance are positively significant, which implies that the SPB-NoHRVB intervention may have positively influenced the swimmers’ perceived ability to cope with training pressures and master the challenges they faced. The psychoeducation phase can explain this result. Indeed, explain to the young athletes how SPB can help to have a cognitive effect in catastrophizing various problems by conveying the idea that various physiological, behavioral, and emotional events can be voluntarily controlled [49]. Then, SPB-NoHRVB can be an interesting coping strategy [27]. Regarding subjective performance, it is possible that completing a breathing task, even without biofeedback, two times a day for three weeks helps the athlete feel more accomplished and competent [27]. The increased perceived control, combined with the psychological effects of SPB-NoHRVB, may also create a virtuous circle in which young athletes feel more able to cope with the demands of training, leading to better overall subjective performance.

The experimental group that underwent the SPB-NoHRVB protocol showed higher biopsychosocial recovery scores than the non-treatment control group. This suggests that the SPB-NoHRVB intervention was effective in promoting a more balanced recovery state among the adolescent swimmers. One explanation may be that perceived control predicts recovery states [50]. Subsequently, the significant improvement in the athlete’s perceived control led to a better perception of total recovery. This effect on the total recovery can also be linked with the psychoeducation given in the first three weeks of the SPB-NoHRVB intervention. Indeed, we emphasized that breathing can enhance recovery. The swimmers then integrated suggestions and theoretical knowledge about recovery and confirmed this knowledge with bodily feedback. In addition, SPB is linked with plenty of psychological/behavioral positive outcomes that can impact subjective recovery (i.e., decreased anxiety, side effects of relaxation, positive energy and pleasantness, and somatic-based emotional control strategies) [25]. During the training phase, young swimmers practiced breathing for 2*10 min a day, which could be enough to make them feel these acute side effects of breathing and improve their subjective experiences, which may modify their interoceptive ability to perceive and interpret changes in body–heart interplays [51]. Finally, recovery can be “passive, active, proactive, and multidimensional” [9]. In the athletic context, passive and active recovery are used predominantly. Indeed, the athlete has little opportunity to take control of recovery strategies, except when it comes to increasing sleep, controlling food intake, and hydration [52]. Coaches and athletes are even encouraged to develop their knowledge about recovery to implement the correct strategies [53]. SPB-NoHRVB allows young athletes the opportunity to take control over their recovery through a multidimensional technique (i.e., heart–brain interplays) and then find clues that indicate they improve recovery. These results are complementary to previous research where an HRV-BFB protocol (i.e., a biofeedback protocol with an individualized breathing rate ranging from 6.5 to 4.5 b/min) showed lower levels of biopsychosocial and cognitive stress for the treatment group compared to a no-treatment control group [54]. No major psychophysiological differences were found between HRV-BFB and no HRV-BFB protocols at 6 b/min [27]. The difference may therefore lie in the amount of SNP activity. Where an SPB-NoHRVB can be closed at the resonant frequency [55], an individualized breathing rhythm is more fine-tuned to reach the 0.1 Hz peak and promote PNS engagement, resulting in lower biopsychosocial and perceived stress. Breathing is also associated with the side effects of relaxation [28]. Therefore, athletes may associate the feeling of being relaxed with an increase in recovery. In short, the SPB-NoHRVB protocol has enabled us to teach and support young athletes to adopt a proactive attitude toward recovery, which has improved their subjective experience of resources (i.e., cognitive secondary appraisal and recovery perception) and performance. In contrast, the lack of significant change in general stress levels within the intervention group, as indicated in Table 1, compared to the control group, might be attributed to an increase in general stress scores over time. This pattern could be linked to heightened recovery awareness resulting from the psychoeducation phase, which emphasized various sources of stress. Such an increase in awareness could lead athletes to identify and report new stressors in different areas. Supporting this notion, Van Daele et al. (2012), in their literature review on the impact of psychoeducation on stress, have demonstrated that the effectiveness of such interventions on stress management can be quite variable [55]. They noted heterogeneity in effects depending on key parameters, with effect sizes in these interventions ranging from −0.10 to 0.78. This review also indicates that psychoeducation appears to be more effective for females. However, in our study, the predominance of male participants in our relatively small cohort could have influenced the outcomes. This gender-related aspect of psychoeducation’s impact adds an additional layer to the complexity of interpreting our results.

In terms of physiological markers, the study found no significant effects of the SPB-NoHRVB intervention on HR recovery and HRV measures. Despite the usefulness and low cost of HRV measurements, which make them a very valuable tool for researchers, a few external parameters can influence HRV. Laborde et al. presented some of them, like oral contraceptives that can influence HRV under stress conditions (e.g., running) or transient variables that can be difficult to control on a long protocol (e.g., a normal sleep routine, no meal or coffee or tea 2 h before the experiment, no alcohol 24 h before the measurements, etc.) [56]. Over seven waves of measurements, most transient variables are impossible to take into account. For example, there was no meal two hours before the experiment. Due to ecological constraints, HRV measurements for the running test were carried out on Saturday morning, one hour before swimming training. It may therefore be difficult to ask young swimmers to skip a meal before their training. Another example is the absence of intense physical training the day before the experiment, which is almost unrealistic given the discipline demands. In terms of HR, daily variations of 6.5% can be observed for submaximal HR and hold for HR recovery [57]. This brings us to the chronic effects of external training load on markers of HR and HRV. For example, endurance training reduces submaximal and resting HR, while the HR rate can be reduced by 3 to 7% [58]. HRV markers also evolve with periodization and training load [59,60]. Consequently, all these physiological disruptions and adaptations may outweigh the potential effects of SPB-NoHRVB on these same markers. Finally, it is well known that slow breathing has an impact on acute markers of HR and HRV [27], but there is a lack of evidence of the same results on long-term effects. A previous study involving 70 healthy non-athlete adults who practiced SPB-NoHRVB for 15 min daily over 30 days demonstrated improvements in sleep quality and cardiac vagal activity [61]. This study, however, ensured strict pre- and post-test conditions, including consistent daily routines, no meals within two hours before bed, and abstaining from alcohol or strenuous physical activity the day before measurements. Replicating such controlled conditions in athletic settings is challenging, as we aim not to interfere with the swim coaches’ periodization plans for the athletes. This discrepancy in the control of external factors and/or the insufficient amount of SPB training could be contributing factors to the observed lack of significant changes in HR and HRV in our study.

The current study has several strengths, such as the use of a well-defined intervention protocol, a randomized controlled design, and the combined assessment of both subjective and objective measures. However, there are noteworthy limitations. The small sample size and our focus on semi-elite adolescent swimmers could narrow the applicability of our results to broader athletic populations. Furthermore, the study’s design did not allow for a gendered analysis due to the small sample, yet understanding gender differences could offer additional insights. A complementary limitation of our study is the use of a non-intervention control group, which, while maintaining ecological validity, may not account for placebo effects that could arise from any form of intervention. Another limitation is the absence of detailed data regarding the specifics of the training load (e.g., aerobic capacity, anaerobic power, etc.). The physiological characteristics of training can significantly influence metabolism and autonomic nervous system responses. Aerobic training, for instance, often enhances parasympathetic activity, leading to increased HRV. Then, we did not measure respiratory frequency during the 5′-5′ test rest period due to ecological constraints, such as the need to test multiple swimmers simultaneously within a limited time frame. This limitation is noteworthy as respiratory frequency can influence HRV indices and may affect the interpretation of physiological responses. Additionally, we collected HRV data weekly; however, a three-weekly collection may provide more nuanced insights [46]. Practical constraints limited the frequency of our measurements. Overall, these nuances in HR and HRV measurements might have impacted our results.

This study builds upon previous work on stress and recovery practices for young athletes [54], demonstrating that SPB-NoHRVB interventions may promote biopsychosocial recovery, enhance perceived control, and improve subjective training performance. Given the limited recovery strategies typically available to young athletes, these interventions could be particularly valuable. Our results are promising and should serve as a foundation for further research, though they should be interpreted cautiously. Despite our non-significant results in HR and HRV, the literature suggests their potential benefits when implemented during training and competition [62]. Future research should focus on increasing the daily duration of SPB practice as well as the length of the training phase (e.g., 3 weeks of daily practice of SPB for 15 min each day), ensuring more controlled conditions before HRV measurements to better assess changes in the PNS. Additionally, considering that suggestions can notably influence cognition and behavior [63], exploring the effects of suggestions during psychoeducation and training, possibly through individual sessions, could yield intriguing findings.

Incorporating our study’s findings, practical applications for coaches and practitioners are evident. Coaches and practitioners can use the SPB-NoHRVB protocol to assist in recovery management. It offers a non-invasive way to improve subjective training performance and perceptions of control. However, it is important to approach these applications cautiously and recognize the need for further research to fully validate these findings and understand their broader implications.

## 5. Conclusions

In conclusion, this study supports the potential benefits of SPB-NoHRVB interventions for adolescent athletes in promoting recovery states, perceived control, and subjective training performance. As a holistic and practical approach, SPB-NoHRVB fits well within the demanding schedules of young athletes. However, considering the limited body of research, the non-significant results regarding HR and HRV, along with other identified limitations, suggest that the outcomes should be interpreted with caution. The technique should be considered as a means to optimize and preserve resources. These insights are crucial in guiding future research to refine these interventions and enhance our comprehension of the long-term effects of SPB-NoHRVB on young athletes.

## Figures and Tables

**Table 1:** Descriptive statistics for the seven waves of measurement.

		Time 0	Time 1	Time 2	Time 3	Time 4	Time 5	Time 6
	Group	M (SD)	M (SD)	M (SD)	M (SD)	M (SD)	M (SD)	M (SD)
sRPE	I	3440.14 (1146.34)	3695 (805.76)	11,474.28 (2668.34)	7868.57 (1050.38)	4930.71 (1421.36)	5365.71 (1072.81)	
	C	3205.98 (1640.05)	4541.67 (745.43)	12,813.33 (1044.08)	6290 (1694.68)	5678.33 (629.11)	5200 (931.63)	
KM	I	24.32 (11.18)	21.63 (4.13)	60.6 (0)	53.11 (8.76)	37.14 (7.16)	35.57 (3.70)	
	C	24.65 (3.16)	24.66 (3.16)	64.8 (0)	55.23 (4.92)	38.07 (4.78)	35.85 (4.84)	
General stress	I	3.25 (1.20)	2.68 (0.95)	4.86 (1.13)	4.97 (1.06)	5.17 (1.01)	4.94 (1.07)	5.11 (1.24)
	C	3.24 (1.56)	2.43 (1.54)	3.55 (1.58)	3.30 (1.57)	2.87 (1.83)	3.30 (1.70)	2.42 (1.18)
General recovery	I	4.14 (0.94)	4.52 (1.13)	4.76 (1.13)	4.97 (1.06)	5.17 (1.01)	4.94 (1.07)	5.11 (1.24)
	C	4.70 (1.56)	5.31 (1.07)	5.26 (1.22)	4.86 (1.90)	4.78 (1.54)	4.87 (1.47)	5.58 (0.89)
Sport-specific stress	I	3.25 (1.15)	2.60 (0.71)	3 (0.98)	2.69 (1.02)	2.03 (0.56)	2.44 (0.88)	2.32 (0.65)
	C	3.14 (0.86)	2.68 (0.84)	3.35 (1.06)	3 (1.17)	2.71 (1.20)	3.24 (1.18)	2.58 (0.66)
Sport-specific recovery	I	3.46 (0.72)	4.68 (0.70)	4.25 (0.49)	4.78 (1.94)	5.11 (0.88)	5.09 (1.17)	4.80 (1.05)
	C	3.89 (1.24)	4.54 (1.28)	4.33 (1.29)	4.46 (1.59)	4.51 (1.18)	3.87 (1.25)	4.71 (1.29)
Total stress	I	3.25 (0.94)	2.64 (0.62)	3.17 (1.11)	2.71 (0.99)	2.14 (0.68)	2.58 (0.90)	2.57 (0.82)
	C	3.20 (1.03)	2.55 (1.01)	3.45 (1.26)	3.15 (1.28)	2.79 (1.38)	3.27 (1.30)	2.5 (1.06)
Total recovery	I	3.80 (0.62)	4.60 (0.73)	4.50 (0.73)	4.88 (0.98)	5.14 (0.87)	5.02 (1.06)	4.96 (1.12)
	C	4.29 (1.37)	4.92 (1.09)	4.80 (1.10)	4.66 (1.73)	4.64 (1.35)	4.37 (1.32)	5.14 (1.06)
Perceived Control	I	3.86 (0.77)	4.38 (0.36)	4.24 (0.46)	4.62 (0.83)	4.90 (0.74)	4.78 (0.72)	4.76 (0.96)
	C	4.17 (0.98)	5 (0.29)	4.5 (1.00)	4.44 (1.07)	4.8 (0.77)	4.44 (0.78)	4.53 (0.77)
Perceived Stress	I	2.42 (1.13)	2 (0.58)	2.38 (0.45)	2.28 (0.78)	1.57 (0.50)	1.89 (0.54)	1.71 (0.62)
	C	2.22 (1.07)	1.05 (0.14)	2.39 (1.14)	2.17 (1.13)	1.6 (0.43)	1.89 (1.09)	1.4 (0.55)
Subjective Training Performance	I	7.71 (4.57)	9.14 (2.85)	9.14 (1.21)	10.14 (2.03)	11 (1.41)	10.83 (2.86)	11.14 (0.90)
	C	9.17 (3.37)	8.67 (3.08)	8.83 (1.33)	9.83 (1.33)	10.4 (1.52)	8.17 (2.56)	8.2 (2.28)
HRex	I	160.6 (12.26)	167.33 (2.52)	153 (9.56)	152.8(11.78)	162 (9.76)	154 (17.78)	
	C	158.4 (9.94)	164 (14.58)	151.17 (7.14)	157.6 (7.10)	159 (5.90)	159.3 (8.73)	
HRR60	I	95.8 (27.29)	84.67 (24.85)	91.2 (19.97)	93.6 (13.70)	109.5 (15.15)	90.67 (23.10)	
	C	83.2 (25.4)	87.8 (25.40)	84.83 (12.66)	83.6 (12.46)	95.83 (21.25)	88.33 (13.90)	
nHRR60	I	0.41 (0.13)	0.49 (0.15)	0.41 (0.10)	0.39 (0.05)	0.32 (0.08)	0.41 (0.11)	
	C	0.47 (0.07)	0.47 (0.12)	0.44 (0.06)	0.47 (0.07)	0.40 (0.11)	0.45 (0.08)	
RMSSD	I	36.28 (35.09)	61.20 (33.45)	63.69 (82.63)	36.50 (30.87)	24.43 (20.69)	34.69 (34.50)	
	C	42.22 (15.07)	49.70 (26.80)	44.05 (22.30)	45.06 (32.35)	35.03 (18.88)	31.87 (14.34)	
LFnu	I	67.17 (17.59)	63.70 (25.57)	54.35 (25.59)	43.65 (20.45)	65.57 (16.23)	56.50 (11.45)	
	C	48.42 (22.80)	60.66 (17.01)	37.87 (13.61)	54.37 (29.99)	65.94 (12.36)	53.99 (22.92)	
HFnu	I	32.79 (17.60)	36.24 (25.50)	45.48 (25.62)	56.16 (20.32)	34.40 (16.21)	43.46 (11.42)	
	C	51.52 (22.83)	39.29 (17.00)	62.04 (13.60)	45.55 (29.92)	33.77 (12.26)	45.84 (23.01)	
LF/HF	I	2.71 (1.66)	2.72 (2.14)	1.68 (1.20)	1.02 (0.87)	2.46 (1.64)	1.46 (0.75)	
	C	1.20 (0.77)	2.16 (1.88)	0.68 (0.38)	2.72 (3.59)	2.37 (1.43)	2.12 (2.56)	
MeanRR	I	705.47 (133.74)	751.29 (92.69)	742.63 (143.99)	744.77 (159.60)	706.41 (124.71)	741.26 (125.94)	
	C	758.32 (61.57)	779.52 (107.96)	777.30 (105.17)	764.85 (105.90)	725.00 (84.53)	739.94 (71.70)	

Note: Time 0 to 6: measurement points of the protocol; Group: I (intervention); C (control); M—mean; SD—standard deviation; sRPE: subjective internal training load; KM: kilometers of training per week; HRex: heart rate exercise; HRR60: heart rate after 60 s of recovery; nHRR60: ratio [(HRex-HR60)/HRex]; LFnu: power of low frequency; HFnu: power of high frequency; LF/HF: ratio of low frequency to high frequency; RMSSD: root mean square of successive RR interval differences; MeanRR: the average R-R interval duration in a measurement.

**Table 2:** The SPB-NoHRVB protocol contents were applied to young swimmers.

Weeks	Session Goals	Tools	Modality
Week 1 (Introduction)	Session 1 (60 min)		Group
	Create adhesion and believe through suggestions Help athletes understand the notion of recovery and stress Introduction of the recovery principles (e.g., active, passive, proactive component, multicomponent aspects). Introduction to the functioning and psychophysiological effects of breathing	Intervention based on a question-and-answer game focusing on athletes’ experiences, knowledge, and beliefs about stress, recovery, and breathing. Use a whiteboard to write down and classify words relating to stress and recovery. Video support and athletes’ participation in breathing	
Week 2 (Skill Development)	Session 2 (60 min)		Group
	Reminders about the previous session Breathing exercises. Starting by discovering how each individual breathes up to SPB Talks about difficulties of breathing if necessary to adjust. Team discussions on the effects of breathing, feelings, and somatic perceptions. Downloading the application “Breath” for the next session	Natural breathing Abdominal breathing Different breathing patterns guided by the mental coach Intuitive slow breathing rhythm Gym mats	
Week 3 (Skill Development)	Session 3 (60 min)		Group
	Reminders and small talks about the previous session Presentation of the coherence technique and advantage for stress and recovery Team discussion on the effects of the coherence effect, feelings, and somatic perceptions. Talks about difficulties. Using and setting up the application	Breath application Gym mats	
Week 4–5 (Home Practice)	Training phase: 2 × 10 min per day		Individually
	Use of the technique Follow-up by the mental coach to ensure that the exercises are completed (go to training, text message to remind participants) Exchange on difficulties if necessary Discuss the psychophysiological and somatic effects they perceive		
Week 6 (Home Practice)	Learning Phase		Group
	Mental coach no longer interferes with the execution or not of the SPB-NoHRVB Observe the effect of the protocol At the end of the 7th week, debriefing on feelings, difficulties, and advantages of the technique. Sharing of experiences		

**Table 3:** Unstandardized parameter estimates of the growth curve in Model 2.

	Performance	Stress–Recovery Balance						Cognitive Appraisal		Heart Rate Recovery and Variability		
	STP	GS	SS	TS	GR	SR	TR	PC	PS	HRex	nHRR60	RMSSD
**Fixed effects—Estimates (Standard errors)**												
**Intercept**	9.34 *** (1.39)	3.03 *** (0.52)	3.05 *** (0.43)	3.04 *** (0.40)	4.96 *** (0.50)	4.24 *** (0.39)	4.60 *** (0.39)	4.51 *** (0.25)	2.02 *** (0.34)	158.52 *** (4.14)	0.47 *** (0.05)	51.69 *** (14.54)
**Time**	−0.08 (0.23)	0.02 (0.06)	−0.01 (0.08)	0.01 (0.06)	0.02 (0.06)	0.01 (0.07)	0.01 (0.06)	0.01 (0.05)	−0.05 (0.06)	−0.11 (0.79)	−0.01 (0.01)	−2.94 (2.57)
**Group**	−1.69 (1.89)	0.14 (0.70)	0.16 (0.58)	0.15 (0.54)	−0.77 (0.68)	−0.45 (0.54)	−0.61 (0.54)	−0.57 (0.34)	0.47 (0.47)	4.22 (5.98)	−0.04 (0.07)	−1.46 (20.96)
Time*Group	0.64 * (0.31)	−0.11 (0.07)	−0.13 (0.11)	−0.12 (0.09)	0.14 (0.09)	0.19 ^¥^ (0.10)	0.16 * (0.08)	0.12 * (0.07)	−0.06 (0.08)	−1.23 (1.14)	−0.01 (0.01)	0.21 (3.73)
**Random effects—Variance (Standard deviation)**												
**σ^2^**	3.15	0.45	0.36	0.34	0.38	0.39	0.28	0.30	0.52	55.11	0.01	617.25
**τ_00subjects_**	9.22	1.27	0.84	0.70	1.20	0.64	0.72	0.16	0.30	47.40	0.01	643.51
**τ_11subjects.time_**	0.19	0.00	0.02	0.01	0.01	0.02	0.01	0.00	0.00	0.22	0.00	0.57
**ρ_01subjects_**	−0.97	0.99	−0.65	−0.16	−0.25	−0.07	0.01	1.00	−0.90	1.00	−1.00	−1.00
**Performance Model**												
**Marginal R²**	0.13	0.02	0.08	0.05	0.04	0.08	0.05	0.12	0.06	0.02	0.12	0.04
**logLik**	−187.58	−108.31	−98.81	−96.99	−103.37	−103.00	−91.45	−83.11	214.1	−219.03	66.149	−290.26

Notes. SE = standard errors; SD = standard deviations; *β*_0*j*_ is the average level of psychological states for individuals; γ_00_ = intercept of level-2 regression predicting *β*_0*j*_; γ_10_ = intercept of level-2 regression predicting *β*_1*j*_; σ^2^ = var(r_ij_) variance in level-1 residual (i.e., variance in r_ij_); τ_00_ = var(U_0j_) variance in level-2 residual (i.e., variance in U_0j_). * *p* < 0.05; *** *p* < 0.001; ^¥^ *p* < 0.10. STP: subjective training performance; GS: general stress; SS: specific stress; TS: total stress; GR: general recovery; SR: specific recovery; TR: total recovery; PC: perceived control; PS: perceived stress; HREx: heart rate exercise; nHRR60: ratio [(HRex-HR60)/HRex]; RMSSD: root mean square of successive RR interval differences.

## Data Availability

Data available on request due to restrictions (e.g., privacy or ethical). The data presented in this study are available on request from the corresponding author. The data are not publicly available because the request was not specified to the ethics committee when the project was submitted.
